# Is a Lead Isotope Ratios in Wine Good Marker for Origin Assessment?

**DOI:** 10.3389/fchem.2021.746695

**Published:** 2021-10-04

**Authors:** Slađana Đurđić, Vesna Stanković, Slavica Ražić, Jelena Mutić

**Affiliations:** ^1^ Department of Analytical Chemistry, Faculty of Chemistry, University of Belgrade, Belgrade, Serbia; ^2^ Department of Chemistry, Institute of Chemistry, Technology and Metallurgy, University of Belgrade, Belgrade, Serbia; ^3^ Department of Analytical Chemistry, Faculty of Pharmacy, University of Belgrade, Belgrade, Serbia

**Keywords:** ICP-QMS, Serbian wines, principal component analysis, geographical origin, lead isotopes

## Abstract

Lead isotope ratio pattern (^206^Pb/^207^Pb, ^208^Pb/^206^Pb, ^206^Pb/^204^Pb, ^207^Pb/^204^Pb, and ^208^Pb/^204^Pb) was analyzed in 59 samples of Serbian wine, from four geographical regions. By utilization of powerful inductively coupled plasma mass spectrometry (ICP-QMS), lead isotope ratios were used as unique “*fingerprint*”, when combined with multivariate methods of analysis (Principal Component Analysis), provided information on the geographical origin of wine. In validation of ICP- QMS method and quantitative analysis, the certified reference material NIST SRM 981 was employed to test the mass-bias correction and thallium isotopes ^203^Tl and ^205^Tl (NIST SRM 997) as an internal standard. The obtained results were discussed in correlation with the corresponding values of LIRs of different European and Australian wines. In addition, the impact of anthropogenic Pb from different sources on the total Pb isotopic composition in Serbian wines was analyzed too. On the other side, the obtained values of Pb content were compared with the applicable health safety standards, according to the International Code of Oenological Practices.

## Introduction

Lead isotopic composition, based on different ratios of stable isotopes (^206^Pb/^207^Pb, ^208^Pb/^206^Pb, ^206^Pb/^204^Pb, ^207^Pb/^204^Pb, and ^208^Pb/^204^Pb), serves as a “*fingerprint*” for definition of different lead sources in the environment (Erel et al., 1997; Larcher et al., 2003; Komárek et al., 2008; Đurđić et al., 2020). Analysis of the lead isotope ratios (LIRs) in food and beverages has provided a powerful tool for determining the geographical origin and authenticity, most often, of wine (Medina et al., 2000; Tian et al., 2000; Barbaste et al., 2001; Mihaljevič et al., 2006; Bora et al., 2018; Epova et al., 2020). In addition, LIRs can be used as a tool for elucidation of chemical environment in a mushrooms-soil system (Đurđić et al., 2020).

Primary, the presence of lead in wines originate from nature, where the vine reflected the isotopic signatures from the geological-pedological environment (natural lead; uncontaminated soli/bedrock). On the other side, lead in wines can also be found as a result of human activities. Anthropogenic lead in wines mainly originates from traffic, fertilizer, pesticide treatment, as well as from metallurgical and smelting activities (Almeida and Vasconcelos, 1999; Đurđić et al., 2020). LIRs might provide information on the origin of the wine assuming that sources of contamination do not distort the original isotope ratio pattern present in the local environment (Tian et al., 2000). ^206^Pb/^207^Pb and ^206^Pb/^208^Pb ratios could be significantly influenced by anthropogenic factors and may be used to differentiate natural and anthropogenic Pb contamination. On the other side, the chemical processes during winemaking have no significant influence on isotopic composition of wine (Rosman et al., 1998). It was reported that the values of the isotope ratios between wines produced in a winery and laboratory were equivalent (Medina et al., 2000). However, the equipment used during the vinification can contribute substantially to the total lead content of the wine and Pb isotope ratios can deviate from those in the soil in the vineyard, which makes authentication rather difficult (Almeida and Vasconcelos, 2004). A precise and accurate lead isotope ratio measurements have been traditionally carried out by thermal ionization mass spectrometry (TIMS). The precision obtainable for isotope ratios determined by inductively coupled plasma mass spectrometry (ICP-QMS) is inherently worse than for many other types of mass spectrometers (Barbaste et al., 2001). For lead isotope ratio (LIR) measurements, the addition of thallium and the measurement of the ^203^Tl/^205^Tl ratio is good way to assess mass bias (Tian et al., 2000). ICP-QMS does not achieve the precision of TIMS but is considered as sufficient to differentiate the origin of wine (Almeida and Vasconcelos, 1999; Larcher et al., 2003; Mihaljevič et al., 2006). Fingerprinting techniques based on chemical composition and multivariate statistical analysis can be used to distinguish the wine origin one from another and to classify them according to region, quality, and type (Kment et al., 2005; Lara et al., 2005; Paneque et al., 2010; Martin et al., 2012).

There are no available data about the LIRs in Serbian wines so, the aim of this paper was to conduct such study (LIRs), to contribute to the databases of LIRs for European wines and to investigate possibility of using it in the discrimination of wine from different regions in Serbia using ICP-QMS.

## Materials and Methods

### Site Description

**FIGURE 1:**
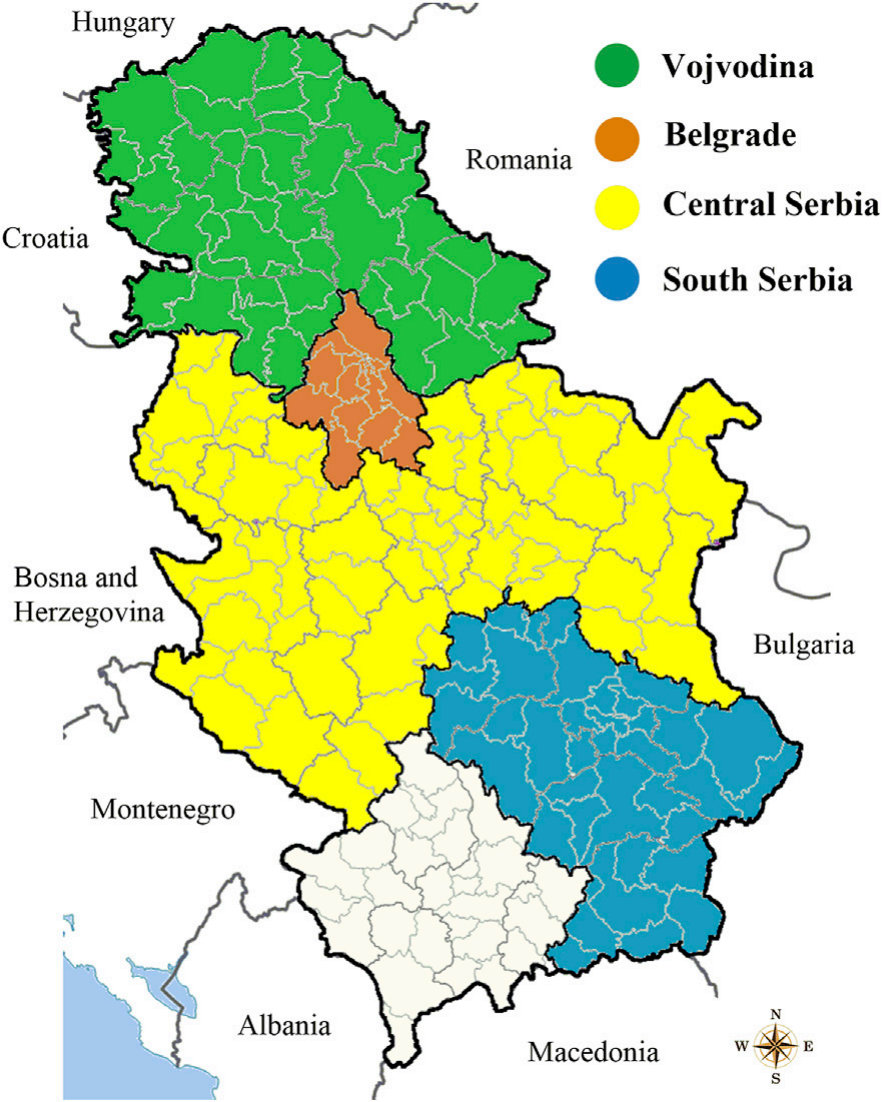
Geographical regions of Serbia for provision of wine samples.

A sample collection of 59 red wines originates from four different regions of Serbia: Vojvodina, Belgrade, Central Serbia and South Serbia. Distribution of wine samples is shown on the map (Figure 1). All wines were supplied in glass bottles with cork stopper.

### Reagents and Chemicals

The certified reference material NIST SRM 981 (Common lead Isotopic standard, National Institute of Standards and Technology, United States) was employed to test the mass-bias correction and for standard solutions. As an internal standard used thallium isotopes ^203^Tl and ^205^Tl NIST SRM 997 (Isotope standard for Thallium, National Institute of Standards and Technology, United States). Mercury stock solution of 1,000 ± 4 μg/ml (TraceCERT, Fluka, Dorset, United Kingdom) was used to study the isobaric interference, originating from ^204^Hg, on ^204^Pb measurements. All glassware was soaked in 10% HNO_3_ for minimum 12 h and rinsed well with ultra-pure water. Ultra-pure water was prepared by passing doubly de-ionized water from Milli-Q system (Millipore Simplicity 185 System incorporating dual UV filters (185 and 254 nm) to remove carbon contamination). All chemicals were of analytical grade and were supplied by Merck (Darmstadt, Germany).

### Instrumentations

Analytical measurements of Pb content and LIRs in wine samples were performed using an inductively coupled plasma mass spectrometer (ICP-MS iCAP Q, Thermo Scientific Xseries 2, United Kingdom) equipped with flat pole collision reaction cell, a micro-concentric nebulizer, nickel cones and a peristaltic sample delivery pump, running a quantitative analysis mode. The entire system controlled with *Qtegra* Instrument Control Software. The use of cooled spray chamber was found to be mandatory in order to minimize the effect of the ethanol matrix. Measured isotopes and instrument operating conditions for determination at ICP-QMS are given in Supplementary Table S1.

### Optimization of Instrumental Parameters for LIRs Analysis

TIMS and ICP sector field multicollector mass spectrometry provides high precision and accuracy during isotope ratios analysis. On the other hand, isotope ratios measurements by ICP-QMS are considered as insufficiently accurate and precise (Vanhaecke et al., 2009), because of the “sequential” measuring approach. Therefore, it is necessary to optimize certain instrumental parameters in order to improve these crucial analytical parameters during the determination of LIRs by ICP-QMS.

The major goal in the isotope ratio measurements by ICP is to achieve constancy of the instrumental mass-bias with the time. Mass-bias effect (mass discrimination) is a feature of all ICP-QMS instruments and occurs due to differential ions transfer from the sample introduction system to the signal detection. Accordingly, ICP-QMS has not the same sensitivity for different masses, due to differences in ion transmission and detection (Devulder et al., 2013). This phenomenon causes a difference between the true values and the obtained LIRs data (Đurđić et al., 2020; Marguí et al., 2007). Therefore, it is mandatory to correct the instrumental LIRs data on the mass-bias effect in order to improve accuracy in LIRs measurements. External and internal standardization can be used for data correction. External correction is based on the use of NIST SRM 981, while NIST SRM 997 was applied for internal standardization (Marguí et al., 2007; Vanhaecke et al., 2009). Also, for the correction of instrumental LIRs data, it is necessary to calculate the bias factor (*K*). For calculation of *K*, a linear, potential or exponential algorithm can be used (Đurđić et al., 2020; Marguí et al., 2007). In Table 1, the values of LIRs of the certified reference material NIST SRM 981 were compared with LIRs instrumental data after their correction to the mass-bias effect, using external and internal standardization with potential algorithm (Đurđić et al., 2020). As noted, external standardization ensured the best compliance of the obtained LIRs of NIST SRM 981 with the certified values of the standard material. Accordingly, this approach was applied to correct all obtained LIRs in wine samples. Finally, the correction of instrumental LIRs data for each wine sample was performed using “bracketing” sequences where the sample was analyzed between two runs of standard solution (Vanhaecke et al., 2009).“Bracketing” was done with standard solution of 5.12 μg/L of lead. In addition, the matrix effect significantly contributes to the mass-bias effect. Matrix effect is dependent on the absolute concentration of matrix components and the most satisfactory methods to eliminate the matrix effect is to remove matrix elements. For that purpose, a large number of chromatographic techniques using chelating or ion exchange column can be used (Jones and Nesterenko, 1997). We tried overcoming matrix effects using the dilution approach due to sample dilution is an easy and effective method to reduce the severity of matrix effects. Several dilutions of the wine sample were tested in order to study the evolution of signal suppression. A dilution factor of 25 demonstrated to be enough to eliminate most of the matrix effects.

**TABLE 1:** Validation of proposed method. Comparison of certified values of NIST SRM 981 with obtained LIRs by ICP-QMS using different correction protocols on mass-bias effect (Đurđić et al., 2020).

LIRs	Certified value ±SD	Found value ±SD^a^ (the correction was performed with external standardization using NIST SRM 981)	Found value ±SD^a^ (the correction was performed with internal standardization using NIST SRM 997)
^207^Pb/^206^Pb	0.91464 ± 0.00033	0.9146 ± 0.0008	0.9191 ± 0.0007
^208^Pb/^206^Pb	2.1681 ± 0.0008	2.1679 ± 0.0009	2.1449 ± 0.0008
^204^Pb/^206^Pb	0.059042 ± 0.000037	0.058 ± 0.003	0.057 ± 0.002

a SD—standard deviation (n = 6).

In addition to correction of LIRs for mass-bias effect, dwell time for each isotope and dead time of detector were optimized in order to increase precision and accuracy of measurements, respectively. These instrumental parameters were optimized by NIST SRM 981 reference material. Standard solution of 5.12 µg/L of lead, spiked with 2 μg/L of thallium, was measured in six points per peak and five replicants, applying different dwell times. Dwell time for each isotope was optimized based on lowest relative standard deviation (RSD) between replicants (Encinar et al., 2001; Marguí et al., 2007). For majority of the analyzed isotopes, RSDs ranged from 0.10 to 0.15%, while RSD for ^204^Pb was 0.32%. Finally, the optimized dwell time was 100 ms for ^204^Pb, 5 ms for ^208^Pb, while the dwell times for isotopes ^206^Pb, ^207^Pb, ^208^Pb, ^203^Tl and ^205^Tl were set to 25 ms. In the case of dead time optimization, Nelms et al. (2001) presents several methods for optimizing this parameter. In this paper, ^204^Pb/^208^Pb was monitored as a function of different lead concentrations (10–30 μg/L), applying different dead times (25, 30, 35, 40 and 45 ns). Dead time of 40 ns was selected as optimal, after linear fitting of the regression line, where the slope of the regression line was close to zero (Nelms et al., 2001; Đurđić et al., 2020). Also, when discussing about the detector of ICP-QMS device, the mode in which the detector operates during LIRs measurements is essential. In this study, all LIRs were analyzed in pulse detector mode. The working range of lead concentration, which provides the operation of the detector of our ICP-QMS device in pulse mode, is up to 40 μg/L. Therefore, a dilution factor of 25 (*Sample preparation*) was sufficient to keep the lead concentration below the critical value, but at the same time, as stated, a dilution of 25 times was sufficient to minimize the matrix effect.

In the end, since ^204^Pb is subject to isobaric interference of ^204^Hg, a correction of ^204^Pb intensity for ^204^Hg was performed (Marguí et al., 2007; Đurđić et al., 2020).

### Sample Preparation

For determination of Pb content, all wine samples were diluted with ultra-pure water (1:10) and directly analyzed. All samples were contained HNO_3_ in final concentration of 2% (v/v). Blank was prepared as standard solutions and wine samples with ultra-pure water and contained HNO_3_ in concentration of 2% (v/v). Further presented Pb concentrations refer to values after blank correction.

For LIRs analysis in wine, samples were diluted with ultra-pure water. Depending of Pb concentration, dilution factor was 10 or 25. This dilution protocol was done in order to provide the pulse mode of the detector, which is mandatory in LIRs analysis. Thallium, as internal standard, was added to all samples in final concentration of 2 μg/L. All wine samples were contained HNO_3_ in concentration of 2% (v/v). Blank was prepared as standard solutions and wine samples, with ultra-pure water, thallium solution and HNO_3_. Final concentration of thallium and HNO_3_ in blank were 2 μg/L and 2% (v/v), respectively. Blank correction has been done for each wine sample.

### Statistical Analysis

Descriptive statistics, correlation analysis and Mann-Whitney *U* test were performed by NCSS software package, www.ncss.com (Hintze, 2001). All data produced were statistically treated to find possible statistically significant differences between the variables with the aid of the nonparametric tests: Kruskal-Wallis and Mann-Whitney U. Principal component analysis (PCA) was carried out by PLS ToolBox, v.6.2.1, for MATLAB 7.12.0 (R2011a).

## Results and Discussion

### Determination of Lead Content in Wine Samples

Basic statistical parameters of Pb analysis, for both of its total content and the isotopic ratios, are summarized in Table 2. The highest average content of Pb was recorded in samples from Vojvodina, while further Pb content decreases in the following order: South Serbia >Central Serbia >Belgrade. In general, the lowest Pb content was 11.50 ± 0.05 μg/L (Central Serbia), while the highest was 126.10 ± 0.09 μg/L (South Serbia). Although a relatively high Pb content was found, it is important to point out that all tested wines are in accordance with the applicable health safety standards: The International Code of Oenological Practices (OIV code, 2019) defines a maximum Pb content in food and beverages of 150 μg/L (this value refers to wines produced since 2007).

**TABLE 2:** Pb isotopic composition and Pb content in red wines from four Serbian regions.

Regions of Serbia	*Statistical parameters*	*Lead isotope ratios*					Pb (μg/L)
		^206^Pb/^204^Pb	^208^Pb/^206^Pb	^207^Pb/^204^Pb	^208^Pb/^204^Pb	^206^Pb/^207^Pb	
**Vojvodina**	*average ± SD* ^a^	**18.3 ± 0.2**	**2.13 ± 0.03**	**16.0 ± 0.2**	**38.9 ± 0.5**	**1.145 ± 0.002**	**65.0 ± 32.6**
	*Min*	17.9	2.10	15.7	38.1	1.111	14.9
	*Max*	18.5	2.16	16.3	39.6	1.168	117.6
**Belgrade**	*average ± SD*	**18.3 ± 0.4**	**2.14 ± 0.03**	**16.0 ± 0.2**	**38.7 ± 0.7**	**1.141 ± 0.003**	**44.4 ± 29.6**
	*Min*	17.6	2.10	15.6	37.4	1.112	12.6
	*Max*	18.6	2.17	16.2	39.8	1.170	100.3
**Central Serbia**	*average ± SD*	**18.3 ± 0.4**	**2.14 ± 0.03**	**16.0 ± 0.3**	**39.0 ± 0.7**	**1.145 ± 0.002**	**48.8 ± 35.1**
	*Min*	17.5	2.10	15.6	37.6	1.112	11.5
	*Max*	18.6	2.17	16.3	39.9	1.171	114.5
**South Serbia**	*average ± SD*	**18.3 ± 0.3**	**2.14 ± 0.02**	**16.0 ± 0.3**	**39.1 ± 0.5**	**1.145 ± 0.003**	**58.0 ± 44.8**
	*Min*	17.9	2.10	15.6	38.0	1.116	14.5
	*Max*	18.6	2.17	16.2	39.9	1.171	126.1

a SD—standard deviation between samples.

**FIGURE 2:**
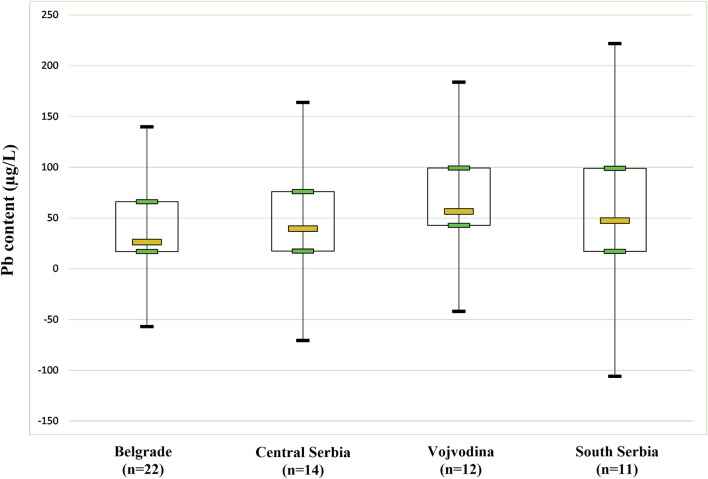
Box and whisker plot of Pb content determined in studied wine samples from different regions of Serbia. Legend: *n*-number of samples.

Figure 2 represents the distribution of lead content in red wine samples from different Serbian regions. Vojvodina, Belgrade, Central Serbia and South Serbia, characterized by medians of 56.4, 26.3, 39.6, and 47.4, respectively. This non-parametric test demonstrates if there are significant differences between the regions.

### Lead Isotope Profile in Serbian Wines

According to our best knowledge, this is the first time that LIRs analysis in Serbian wines has been done. The average LIRs (average ± SD) by their geographical origin are presented in Table 2. Small differences among the wines from the Serbian regions were observed for the ^208^Pb/^206^Pb, ^206^Pb/^207^Pb, and ^208^Pb/^204^Pb ratios. On another hand, average values of ^207^Pb/^204^Pb and ^206^Pb/^204^Pb were very similar in all Serbian regions. Minimal changes occur due to the fact that isotope ^207^Pb changes slowly in time, in comparison with ^208^Pb and ^206^Pb isotopes, since ^235^U generally decayed, while the abundance of ^232^Th and ^238^U is still relatively high on Earth (Komárek et al., 2008).

**FIGURE 3:**
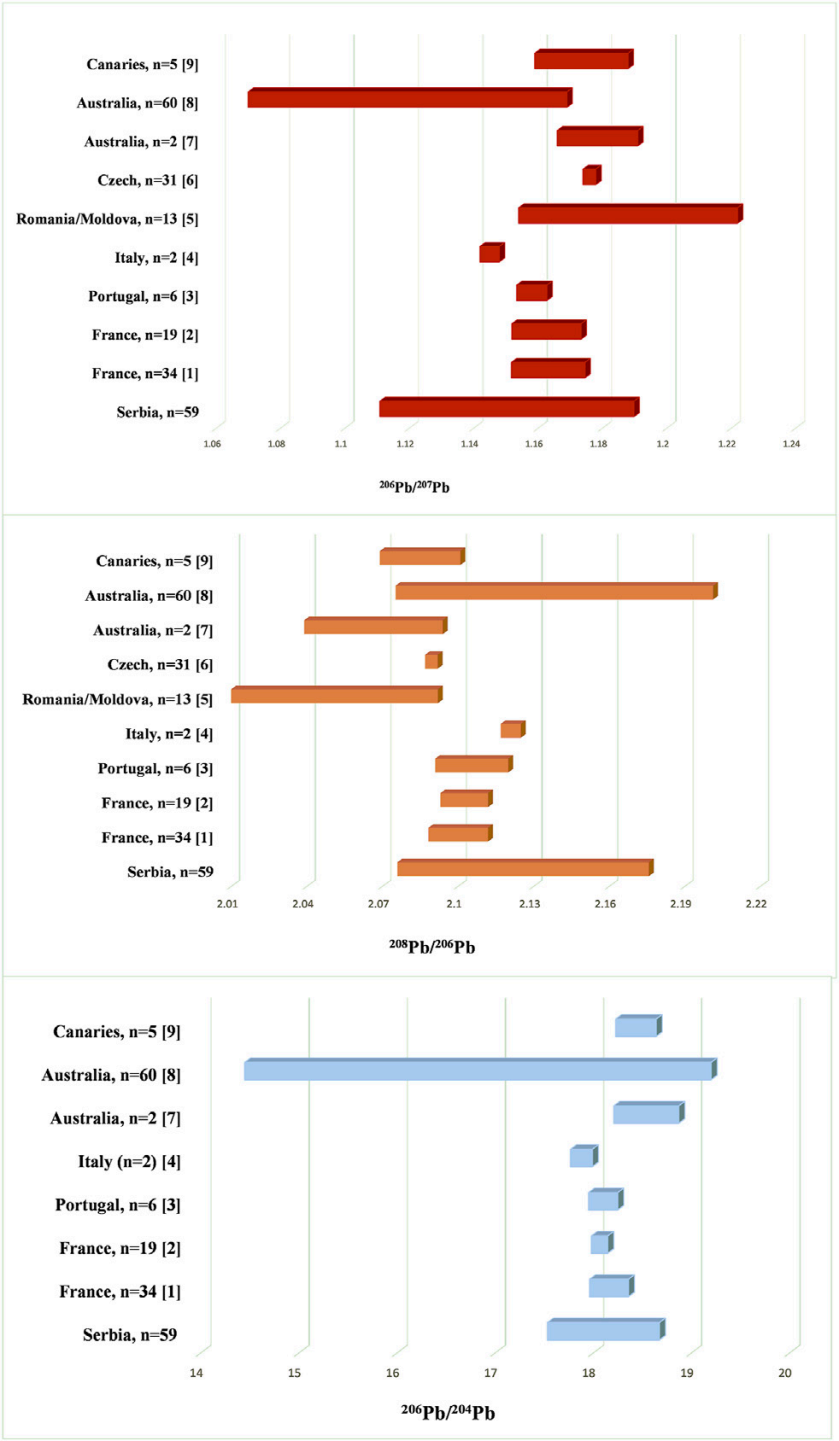
The ranges of the values of ^208^Pb/^206^Pb, ^206^Pb/^207^Pb and ^206^Pb/^204^Pb for wines from different geographical areas, reported in literature. References: (1) Epova et al. (2020); (2) Rosman et al. (1998); (3) Almeida and Vasconcelos (1999); (4, 7, 9) Tian et al. (2000); (5) Dehelean and Voica (2012); (6) Mihaljevič et al. (2006); (8) Kristensen et al. (2016).

The obtained results showed that Serbian wines covered central part of the ^208^Pb/^206^Pb, ^206^Pb/^207^Pb and ^206^Pb/^204^Pb ratios and are in concordance with LIRs of different European wines (Figure 3). In this case, ranges of ^208^Pb/^206^Pb, ^206^Pb/^207^Pb, and ^206^Pb/^204^Pb ratios were from 2.0760 to 2.1758, 1.1109 to 1.1898 and 17.53 to 18.67, respectively. Ranges of ^208^Pb/^206^Pb and ^206^Pb/^204^Pb ratios of Serbian wines were also within the wide ranges of Australian red wines (^208^Pb/^206^Pb - from 2.0753 to 2.2013; ^206^Pb/^204^Pb—from 14.45 to 19.20) reported by Kristensen et al. (2016), while values of ^206^Pb/^207^Pb of our wines were slightly higher than reported (^206^Pb/^207^Pb—from 1.0700 to 1.1690). On the other hand, Australian and Canaries wines reported by Tian et al. (2000) slightly coincide with the observed LIRs of Serbian wines. The different Pb isotopic composition of the mentioned wines in relation to Serbian wines potentially indicates a different source of Pb pollution in the observed regions (Komárek et al., 2008). On the other hand, Romanian/Moldavian wines were characterized by very low ^208^Pb/^206^Pb range (1.9961–2.0920) and a significantly high range of ^206^Pb/^207^Pb (1.1540–1.2220) compared to Serbian wines. Such Pb isotopic composition in Romanian/Moldavian wines is suspected to coal combustion whose isotope ratios values are similar to natural lead (Komárek et al., 2008; Dehelean and Voica, 2012).

### Tracing the Geographical Origin of Wines by Non-Radiogenic ^204^Pb Isotope

In order to define the geographical origin of wine, it is necessary to consider the geogenic ^204^Pb. According to *a priori* knowledge and environmental research, the ^204^Pb isotope defines natural Pb-sources in environmental samples (Epova et al., 2020). In that sense, the content of ^204^Pb in wines shows the isotopic composition in wine, and the corresponding soil, where the grape was grown, since the plant inherits isotopic signatures from the geological-pedological environment. In this way, the authenticity of the wine can be established (Martins et al., 2014; Bora et al., 2018; Epova et al., 2020).

**FIGURE 4:**
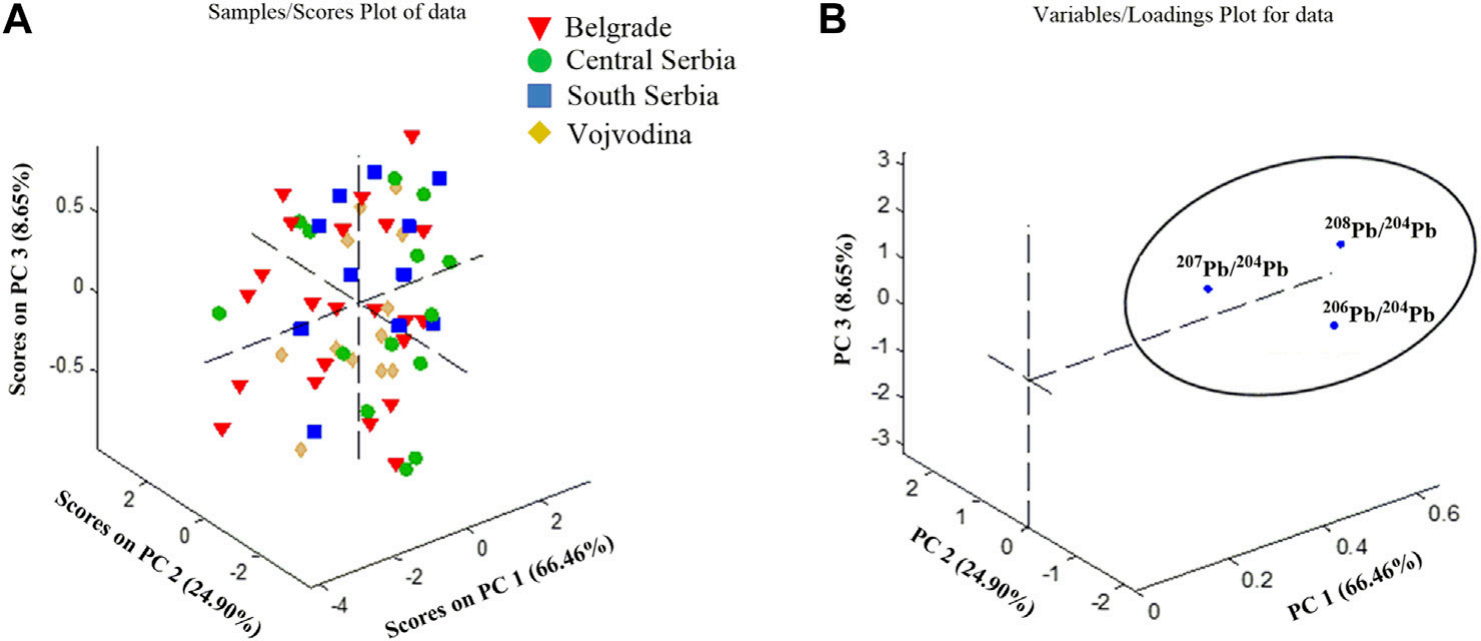
PCA model for distribution of LIRs in Serbian wines; **(A)** a score plot; **(B)** loading plot.

For this purpose, the content of ^204^Pb was discussed and correlated with the radiogenic isotopes ^208^Pb, ^207^Pb and ^206^Pb. The corresponding LIRs of all four Serbian regions (^208^Pb/^204^Pb, ^207^Pb/^204^Pb, and ^206^Pb/^204^Pb) were subjected to the Principal Component Analysis (PCA). All data were autoscaled prior to any multivariate analysis to bring values to compatible units. PCA was carried out as an exploratory data analysis by using a singular value decomposition algorithm and a 0.95 confidence level for Q and T2 Hotelling limits for outliers. The analysis was based on correlation matrix and factors with eigenvalues greater than one were retained. Sample scores and their loadings for the three PCs (PC1, PC2, and PC3), responsible for 100% of total variance, are presented in Figure 4. With a score plot (Figure 4A), the complete overlap of all regions of Serbia was obtained, while the loading graph (Figure 4B) clearly indicates the grouping according to all analyzed LIRs. It means that no relevant discrimination of wines according to their geographical origin was observed.

**FIGURE 5:**
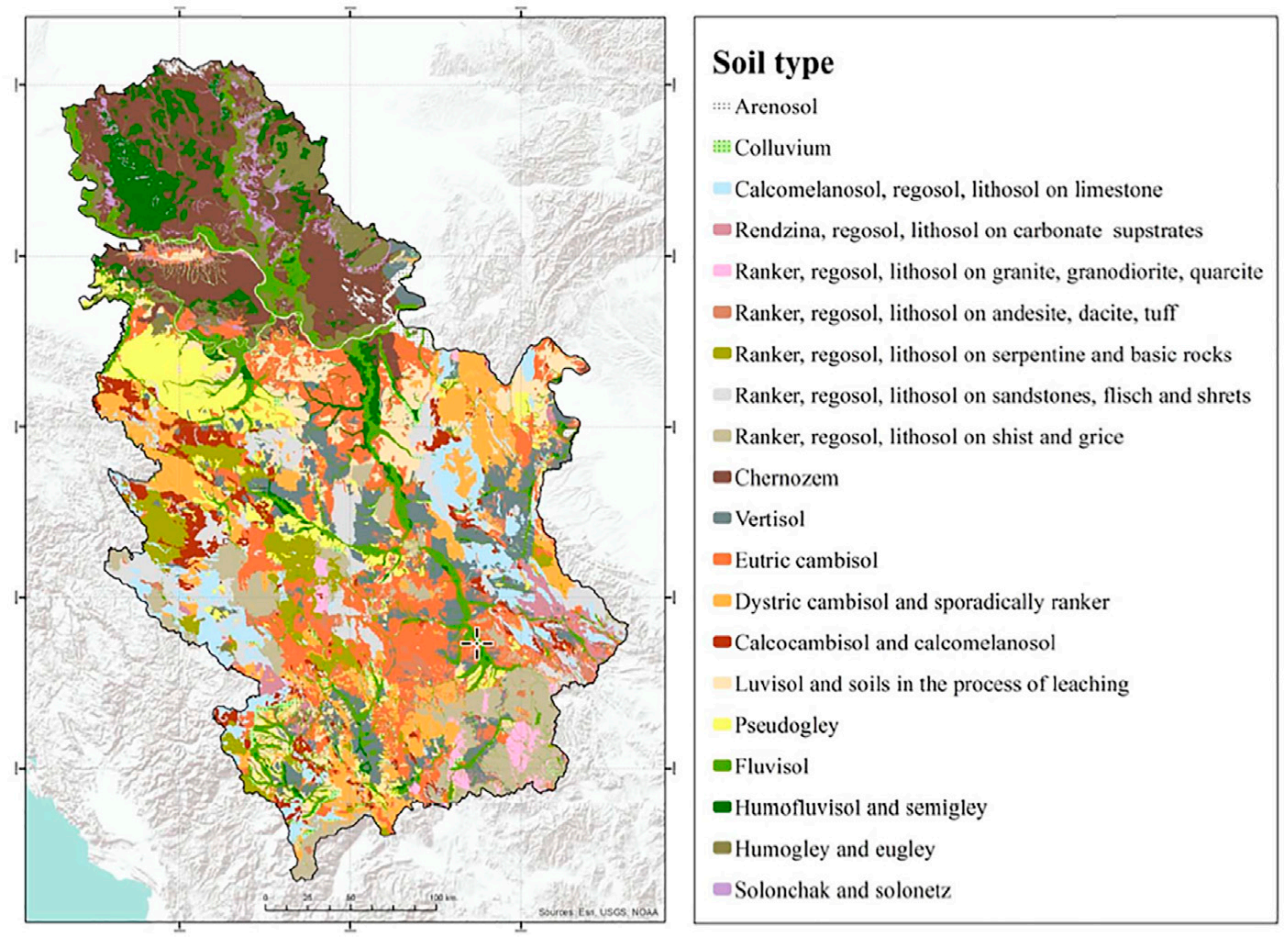
The map of Republic of Serbia according to the type of soil (Čakmak et al., 2018).

The map of the Republic of Serbia (Čakmak et al., 2018) shows different soil profile in regions (Figure 5). It is clear that Vojvodina, as the largest Serbian plain, is characterized by chernozem and humofluvisol. In the area of Belgrade, eutric cambisol and luvisol soil types are dominant. Central and South Serbia, as a hilly and mountainous segment of Serbia, is characterized by diverse soil type such as vertisol, eutric cambisol, calcomelanosol and ranker. Among other factors, different soil types reflect different content of macro and micro elements (including Pb), as result of different processes in pedogenesis (Čakmak et al., 2018; Vukojević et al., 2019). Since the content of ^204^Pb in wines (in combination with other LIRs) reflects the isotopic composition in the corresponding soil, the recognition of Serbian regions is foreseen. However, anthropogenic sources can influence the Pb isotopic composition and should not be neglected.

### Tracing Anthropogenic Influences Using ^208^Pb/^206^Pb and ^206^Pb/^207^Pb

In environmental studies, plotting of ^208^Pb/^206^Pb vs.^206^Pb/^207^Pb is used to identify different sources of Pb contamination such as natural, geogenic Pb (rock and soil formation) and anthropogenic (emissions from cars, industrial existence, metal activities, smelters … ) (Erel et al., 1997; Larcher et al., 2003; Komárek et al., 2008; Đurđić et al., 2020).

**FIGURE 6:**
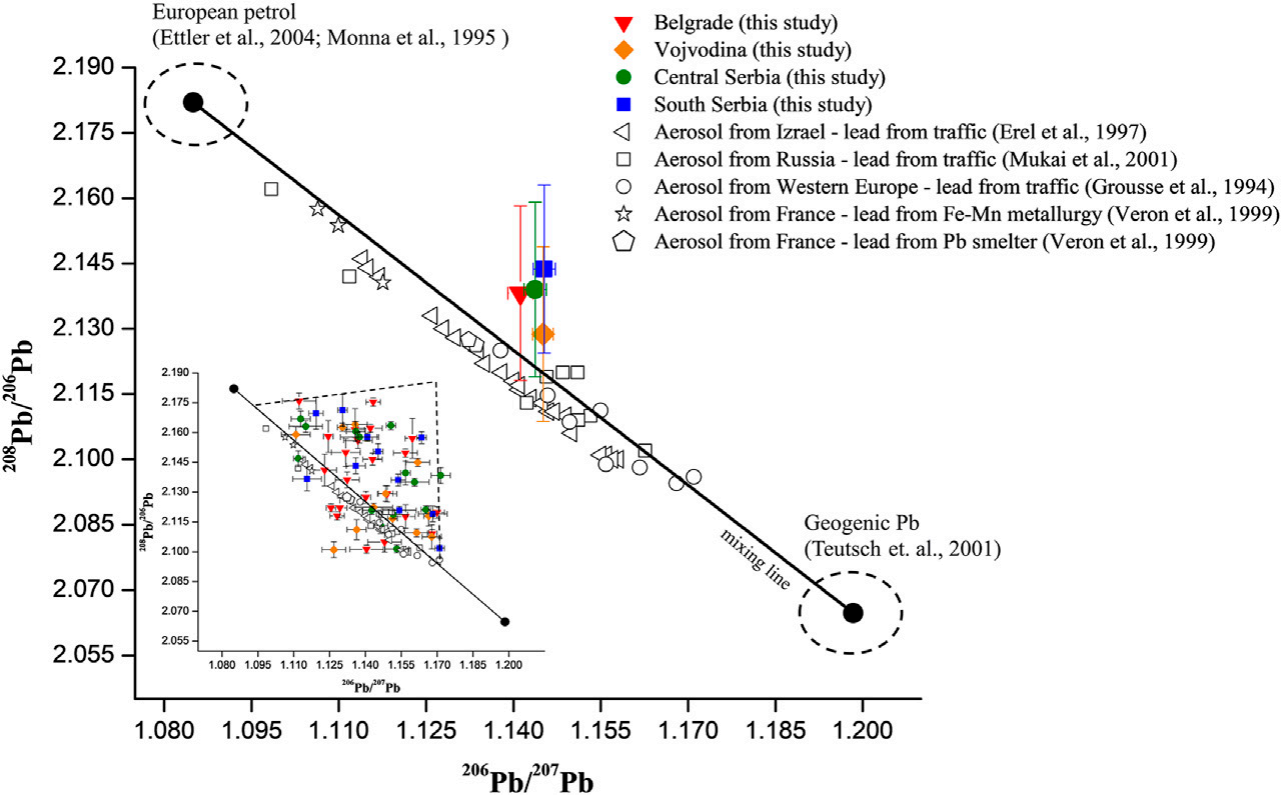
Plot of average ^208^Pb/^206^Pb and average ^206^Pb/^207^Pb of analyzed Serbian wines. Error bars represent standard deviations between samples. Comparison with identical LIRs in aerosols from different European countries. Inset figure represent distribution of ^208^Pb/^206^Pb and ^206^Pb/^207^Pb for individual wine samples from four Serbian regions.

Lead particles emitted into the atmosphere are mostly retained in the atmosphere (atmospheric Pb), exhibiting Pb isotopic signature of atmospheric aerosol (Flament et al., 2002). It was reported that the average size of the anthropogenic Pb particle was about 0.9 µm, although the size of the emitted Pb particles varies depending on the source of pollution. The problem with contaminated aerosol occurs due to uncontrolled aerosol transport as a consequence of various climatic factors. This leads to difficulties to define Pb isotopic profile, as well as to identify sources of Pb pollution (Komárek et al., 2008). According to the reported data, the dust particles, originating from the Sahara, contribute significantly to the Pb isotopic composition in the European aerosol (Doucet and Carignan, 2001; Grousset et al., 1994). Erel et al. (2002), analyzing LIRs in atmospheric aerosol in Jerusalem (Israel), found a significant contribution of Pb that does not originate from a local source, but from other parts of the World (Eastern Europe, Turkey and Egypt). For example, LIRs in French wine from 19 different vintage (1950–1991), were influenced by contribution of Pb variations in aerosol (Rosman et al., 1998). Kristensen et al. (2016) defined the LIRs in Australian wines in correlation to leaded gasoline, especially during the 1960s and 1970s. The authors explained that influence of Pb in the atmospheric aerosol significantly contributed to the total Pb isotopic composition in wine. Besides, clearly separation from the LIRs obtained for the vineyard soil was observed (Kristensen et al., 2016). In this study, we compared average values of LIRs of Serbian wines from different regions with literature data (Erel et al., 1997; Grousset et al., 1994; Kristensen et al., 2016; Mukai et al., 2001; Véron et al., 1999) related to atmospheric aerosol containing Pb from different anthropogenic sources (leaded gasoline from different regions/countries, Fe-Mn metallurgy, Pb smelter) and presented on Figure 6. Three-isotopes “mixing line” (^208^Pb/^206^Pb vs.^206^Pb/^207^Pb), obtained between the geogenic (Teutsch et al., 2001) and the anthropogenic LIRs, characterizes European petrol (as the most common anthropogenic source of Pb) (Ettler et al., 2004; Monna et al., 1995). The graph shows a very good accordance of the “mixing line” with LIRs characterized by Pb from traffic, Fe-Mn metallurgy and Pb smelter. LIRs of Serbian wines were distributed along the “mixing line” (inset Figure 6), what is in accordance with the literature data (Erel et al., 1997; Grousset et al., 1994; Kristensen et al., 2016; Mukai et al., 2001; Véron et al., 1999). From Figure 6, the average values of LIRs for four Serbian regions are proximately equivalent to one another and showed a typical value of the mixture of European petrol and geogenic Pb. This indicates that Pb in the aerosol from different sources significantly contributed to the total Pb isotopic composition in Serbian wines. On the other hand, a scattering of LIRs data of Serbian wines from “mixing line”, in the shape of an imaginary triangle, was observed (inset Figure 6). Such scattering was described by Vanhaecke et al. (2009), explaining that Pb isotopic composition in environmental samples is, in majority of cases, defined by more than two Pb sources. Unknown or uncategorized sources of pollution have to be considered as well.

The vinification process is also one of the important sources of lead contamination of wine. The vinification process begins with the grape harvest and continues with the transport of grapes to the winery, the crushing of grapes and the addition of pectolytic enzyme preparations for maceration and antioxidants (ascorbic acid and tannin). These processes were followed by the addition of wine yeast to the must, filtration of the must, as well as sulfurization with certain doses of SO_2_-based compounds to final products in bottles. All these vinification steps represent potential sources of lead in wine (Almeida and Vasconcelos, 2003; Stockley et al., 2003). Recently, with the massive introduction of stainless-steel devices the contamination level was drastically reduced, but still, during traditional vinification protocol, a long contact of wine with Pb equipment significantly contributes to Pb isotope composition in wine (Almeida and Vasconcelos, 2003). This influence is especially pronounced in the preparation of red wine, since the maceration process is one of the main processes in its production. We believe that Pb from the vinification process, in addition to leaded gasoline, metallurgical activities/smelters, also contributed to the total Pb isotopic composition in wines. On the other side, this synergistic effect of all those influences cause difficulties to define Serbian wine regions.

**FIGURE 7:**
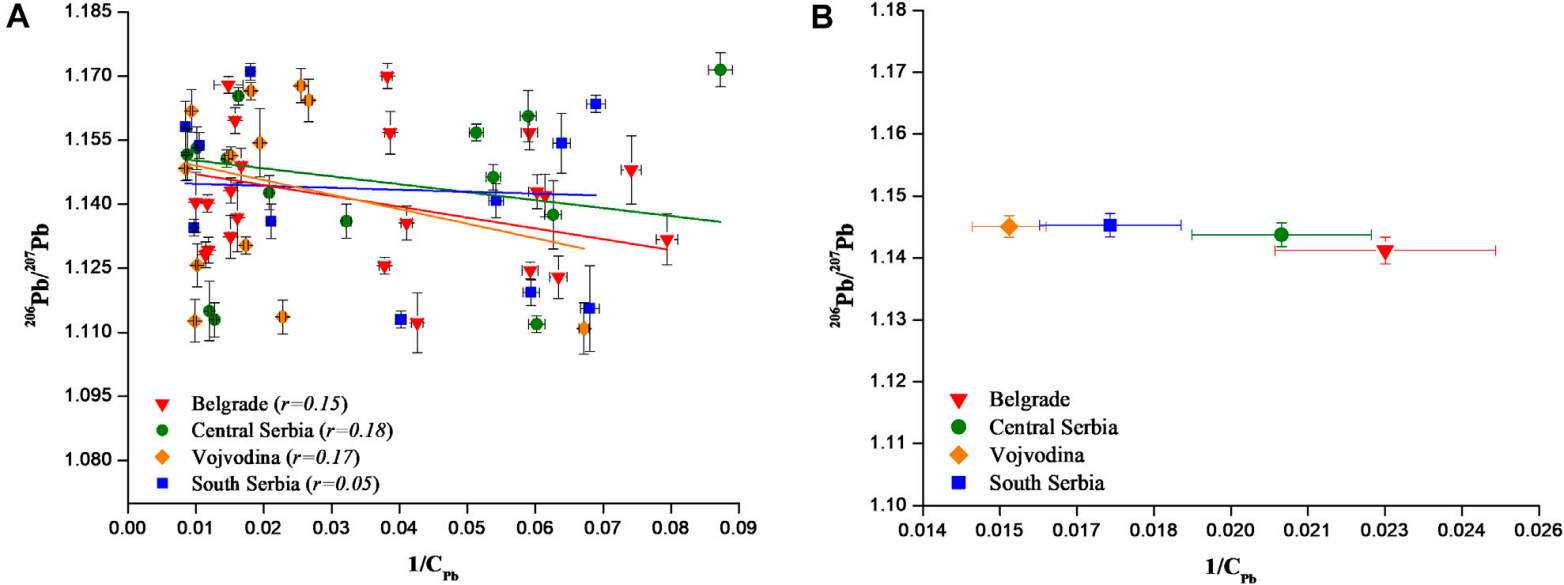
**(A)** Possible isotopic signature of pollutant Pb sources in Serbian wines determined from ^206^Pb/^207^Pb ratio vs. 1/C_Pb_. **(B)** Plot of average ^206^Pb/^207^Pb ratio vs. average 1/C_Pb_ for four Serbian regions. Error bars represent standard deviations between samples.

In a final step, the isotope ratio ^206^Pb/^207^Pb was correlated with 1/C_Pb_ (C_Pb_—Pb concentration) and the obtained results graphically presented on Figure 7A. When statistically reliable, the extrapolation of 1/C_Pb_ toward 0 provides the isotopic signature of the source of contamination, assuming it is a single source with a constant isotopic composition (Erel et al., 1997; Ndung’u et al., 2011). However, the Pearson correlation coefficient (r) for analyzed wines from four Serbian regions was from 0.05 to 0.18. Such a correlation indicates that there is no statistically significant difference between ^206^Pb/^207^Pb and 1/C_Pb_ due to the large variance of the data, indicating possible multiple sources of lead pollution (Ndung’u et al., 2011). This is also consistent with data discussed and presented on Figure 6. In addition, plot of average ^206^Pb/^207^Pb vs. average of 1/C_Pb_ for all Serbian regions (Figure 7B) showing that the LIRs are independent of Pb concentration.

## Conclusion

In this work, systematic analysis of lead isotope profile in Serbian wines from four regions was conducted. The obtained results are discussed in order to identify potential sources of lead pollution and compared with those for European and Australian wines. The obtained values of the ^208^Pb/^206^Pb, ^206^Pb/^207^Pb, and ^206^Pb/^204^Pb ratios of Serbian wines are comparable to ratios reported for other European areas and contribute to the databases for lead isotope profiles of European wines. Further evaluation showed that, in addition to natural Pb, various anthropogenic sources such as leaded gasoline, Fe-Mn metallurgy, Pb smelter contribute to the total Pb isotopic profile of Serbian wines. Besides, the vinification process is also suspected to have a significant influence.

LIRs are powerful tool for tracing the origin of wine on condition that a single source of influence i.e., pollution is previously confirmed. In the case of multiple sources of influence isotopic signatures are not significantly different due to large variance of data. In any case, both fully validated ICP-QMS analysis and overall methodology, coupled with multivariate method of analysis (PCA) offer the best approach to answer the entitled question of tracing the origin of wine, determining the authenticity and geographical origin of wine.

## Data Availability

The original contributions presented in the study are included in the article/Supplementary Material, further inquiries can be directed to the corresponding author.
