# Supplementary material for: Is a Lead Isotope Ratios in Wine Good Marker for Origin Assessment?
Source: Front Chem. 2021 Oct 4;9:746695. doi: 10.3389/fchem.2021.746695 (PMC8521031; doi:10.3389/fchem.2021.746695)
Supplement: Supplementary file 1 [file DataSheet1.PDF]

Supplementary Material

1 Supplementary Tables

Supplementary Table 1. Instrument operating conditions for determination of Pb content and LIRs in Serbian wine samples

ICP-QMS operating conditions	
Rf power (W)	1548
Gas flows (L/min)	13.90; 1.09; 0.80
Acquisition time	3 x 50 s
Points per peak	3
Sample uptake rate (mL/min)	0.4
No. of replicates	6
Detector mode	pulse
Measured isotopes	^{204}Pb, ^{206}Pb, ^{207}Pb, ^{208}Pb, ^{203}Tl, ^{205}Tl, ^{204}Hg, ^{202}Hg
